# Clinical Studies on Cytokine-Induced Killer Cells: Lessons from Lymphoma Trials

**DOI:** 10.3390/cancers13236007

**Published:** 2021-11-29

**Authors:** Ying Zhang, Amit Sharma, Hans Weiher, Matthias Schmid, Glen Kristiansen, Ingo G. H. Schmidt-Wolf

**Affiliations:** 1 Department of Integrated Oncology, Center for Integrated Oncology (CIO), University Hospital Bonn, 3127 Bonn, Germany; Ying.Zhang@ukbonn.de (Y.Z.); Amit.Sharma@ukbonn.de (A.S.); 2 Department of Neurosurgery, University Hospital Bonn, 53127 Bonn, Germany; 3 Department of Applied Natural Sciences, Bonn-Rhein-Sieg University of Applied Sciences, 53359 Rheinbach, Germany; Hans.Weiher@h-brs.de; 4 Institute for Medical Biometry, Computer Science and Epidemiology, University Hospital Bonn, 53127 Bonn, Germany; matthias.c.schmid@uni-bonn.de; 5 Institute of Pathology, University Hospital Bonn, 53127 Bonn, Germany; glen.kristiansen@ukbonn.de

**Keywords:** lymphoma, cytokine-induced killer cells, clinical study, immunotherapy

## Abstract

**Simple Summary:**

Lymphoma is a heterogeneous group of neoplasms including over 70 different subtypes. Its biological characteristic of deriving from lymphoid tissues makes it ideal for immunotherapy. In this paper, we provide insights into lymphoma-specific clinical trials based on cytokine-induced killer (CIK) cell therapy. We also reviewed pre-clinical lymphoma models where CIK cells have been used along with other synergetic tumor-targeting immune modules to improve their therapeutic potential. From a broader perspective, we will highlight that CIK cell therapy has potential, and in this rapidly evolving landscape of cancer therapies its optimization (as a personalized therapeutic approach) will be beneficial in lymphomas.

**Abstract:**

Cancer is a complex disease where resistance to therapies and relapses often pose a serious clinical challenge. The scenario is even more complicated when the cancer type itself is heterogeneous in nature, e.g., lymphoma, a cancer of the lymphocytes which constitutes more than 70 different subtypes. Indeed, the treatment options continue to expand in lymphomas. Herein, we provide insights into lymphoma-specific clinical trials based on cytokine-induced killer (CIK) cell therapy and other pre-clinical lymphoma models where CIK cells have been used along with other synergetic tumor-targeting immune modules to improve their therapeutic potential. From a broader perspective, we will highlight that CIK cell therapy has potential, and in this rapidly evolving landscape of cancer therapies its optimization (as a personalized therapeutic approach) will be beneficial in lymphomas.

## 1. Introduction

Contrary to solid cancers, hematologic malignancies (blood cancers) have some unique characteristics and often require different treatments. Importantly, it is now well established that the hematologic malignancies (lymphoma, leukemia, and myeloma) despite sharing some common clinical features are also biologically distinct entities. Among them, lymphoma, a cancer of lymphocytes, is a very heterogeneous group of neoplasms exhibiting diverse clinical presentations, prognoses, and therapeutic responses and includes more than 70 different subtypes. Lymphomas are conventionally divided into two main groups, Hodgkin’s lymphoma (HL) and non-Hodgkin’s lymphoma (NHL), with both types having multiple early clinical manifestations, often delaying the diagnosis. By comparison, HLs are rare and less heterogeneous, whereas NHLs are more a common and highly heterogeneous group of B-, T-, or NK-cell neoplasms. HL only accounts for approximately 10% of newly diagnosed lymphoma [1]. The complex heterogeneity can also be evident from the fact that in HL alone, the distinctions can be made between two subtypes, classic HL (cHL) and rare nodular lymphocyte-predominant HL (nLPHL) [2]. Besides, cHL is further categorized into nodular sclerosis (NS), mixed cellularity (MC), lymphocyte-rich (LR) and lymphocyte-depleted (LD) cHL. The cHL subtype is composed of Hodgkin (H) and Reed-Sternberg (RS) cells, collectively referred to as Hodgkin and Reed-Sternberg cells (HRS). The scenario is more complex in NHL, which is classified according to the type of lymphocytes involved: B lymphocytes (B-cells) or T lymphocytes (T-cells). Diffuse large B-cell lymphoma (DLBCL), follicular lymphoma (FL), and Burkitt’s lymphoma (BL) are common NHL types that are usually classified according to their aggressive or slow growing characteristics.

As per reports from the SEER database (https://seer.cancer.gov/, accessed on 3 August 2021), HL affected approximately 219,128 people in the United States in 2018. To mention, HL is quite curable, but there are differences in incidence rates, sex and age distribution (young and adults), mainly based on the socioeconomic background [3]. The most common age group of diagnosis is generally between 20 and 34 years old. Contrarily, NHL pose a challenge to the treatment response. According to the German Center for Cancer Registry Data (ZfKD), about 19,200 people in Germany were diagnosed with NHL in 2017, mostly in people of advanced age (average: women 72 years, men 70 years). In 2020, the number of incident cases is increased to 18,549 in Germany from the data of GLOBOCAN 2020 (http://gco.iarc.fr/today, accessed on 25 November 2021). Similarly, approximately 81,560 patients are expected to be diagnosed with NHL in the United States in 2021 according to data from Cancer Statistics Center of American Cancer Society (http://cancerstatisticscenter.cancer.org/, accessed on 3 August 2021).

**Figure 1:**
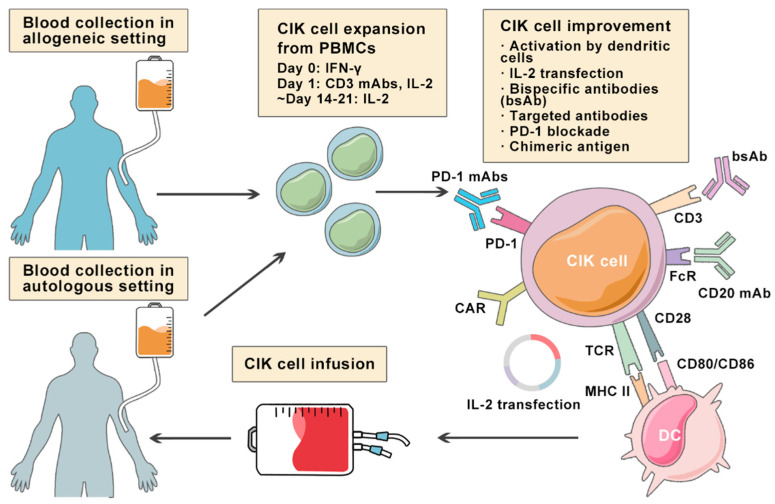
Overview of multiple approaches aiming at improving cytokine-induced killer (CIK)cell therapy in lymphoma. Illustration showing the expansion of autologous or allogeneic CIK cells from PBMCs collected from the blood of lymphoma patients under incubation of IFN-γ, CD3 mAbs, and IL-2 for 2–3 weeks. To increase the cytotoxicity and specificity of CIK cells against lymphoma cells, several modifications are implemented.

Herein, we provide insights into lymphoma-specific clinical trials based on cytokine-induced killer (CIK) cell therapy and other pre-clinical lymphoma models where CIK cells have been used to improve their therapeutic potential (Figure 1).

## 2. Current Immunologic Approaches in Lymphoma Therapy

Being heterogeneous in nature, lymphomas also exhibit heterogeneity in the tumor microenvironment, which reflects in differential response to the therapy. Particularly in the aggressive NHL types, how these malignant cells can mask and protect themselves from immune surveillance holds the key to future treatment strategies. On a broader prospective, the use of the immune system to treat lymphoma has offered an attractive alternative and continues to be an opportunity for improvement in treatment, e.g., through antibody therapy (alone or in combination with chemotherapy as chemo-immunotherapy), checkpoint inhibitors, chimeric antigen receptor T (CAR-T) cell therapy, allogeneic hematopoietic cell transplantation (alloHCT) and Antibody-drug conjugates (ADCs) [4].

Concerning antibody therapy, the results of the monoclonal antibody against CD20 (rituximab) showed very promising results in B-cell lymphomas [5,6]. The combination of anti-CD20 antibody with other antibodies (anti-CD80 and anti-CD22) has proven to be effective in follicular lymphoma [7,8]. Immunochemotherapy with the anti-CD20 monoclonal antibody rituximab (R) in combination with CHOP (cyclophosphamide, doxorubicin, vincristine, prednisone) did not show superiority compared to obinutuzumab (G-CHOP) in diffuse large B-cell lymphoma (DLBCL) [9]. In fact, bispecific monoclonal antibodies have shown promising responses, particularly in patients with relapsed and refractory B-cell lymphoma (CD3-CD20) [10]. Here, it is important to mention the bispecific T-cell engager (BiTE) antibody blinatumomab (CD3-CD19) in relapsed/refractory diffuse large B-cell lymphoma, which has been shown to be effective in heavily pretreated patients with substantial medical needs [11]. Among checkpoint inhibitors, blockade of the PD-1/PD-L1 axis induced significant responses in diffuse large B-cell lymphoma, follicular lymphoma, and various T-cell lymphomas [12].

CAR T-cell therapy is being actively investigated in all hematologic malignancies and is showing promising results in Hodgkin lymphoma [13] and follicular lymphoma [14]. Of note, alloHCT previously had a definite role in the treatment of NHL and still has a perspective in the rapidly evolving treatment landscape of DLBCL, FL, MCL, and PTCL [15]. Here, it is also important to mention Antibody-drug conjugates (ADCs) that have been approved and are active in lymphoma treatment [16]. Among them, brentuximab vedotin (BV), an antibody-drug conjugate directed against the CD30 antigen, has shown encouraging results in patients with relapsed classical Hodgkin’s lymphoma (cHL) and relapsed anaplastic large cell lymphoma (ALCL) [17].

## 3. Cytokine-Induced Killer Cells

Since cancer immunotherapy focuses on enhancing the anti-cancer capabilities of immune cells rather than killing cancer cells, one such potential approach of adoptive immunotherapy is cytokine-induced killer (CIK) therapy, which eliminates cancer cells by transfusing immune cells that have been expanded and activated in vitro. Phenotypically, CIK cells are heterogeneous in nature and composed of CD3+CD56− (T cells), CD3−CD56+ (NK cells), and CD3+CD56+ (NKT cells) cell populations [18]. In fact, the CD3+CD56+ subset is considered as the major effector cells that exert potent anti-tumor cytotoxicity in a major histocompatibility complex (MHC)-unrestricted manner. The generation of CIK cells was first reported in 1991 by sequential incubation of peripheral blood mononuclear cells (PBMCs) with interferon-γ (IFN-γ), anti-CD3 monoclonal antibody (OKT3), and recombinant human interleukin-2 (rhIL-2) [19]. The manufacturing process of autologous and allogeneic CIK cells is shown in Figure 1.

Particularly, apparent differences can be drawn between CIK cells and the impressive immunologic approaches against lymphoma, including CAR-T and BiTE therapies (Table 1). Unlike CAR-T and BiTE that recognize tumor cells by targeting the tumor-associated antigens (TAAs), the majority of the CIK cell cytotoxicity results from the natural killer group 2 member D (NKG2D) interactions [20,21,22,23]. NKG2D is expressed on all NK cells and recognizes at least six ligands that are relatively restricted on malignant tissues, including MHC class I-related molecules A and B (MICA and MICB) and members of the UL16-binding protein family (ULBP1-4) [24]. The manufacturing of CAR-T cells requires genetic modification and cell expansion ex vivo, which is time-consuming and brings some financial considerations. However, the relative easiness of CIK cell preparation facilitates the fast growing of CIK cell therapy. Besides, cytokine release syndrome (CRS) is the most severe toxicity in CAR-T and BiTE therapies. It is associated with the rapid and extensive activation of T cells and the release of high-level proinflammatory cytokines (TNFα and IL-6) [25]. Nonetheless, the crucial advantages of CIK cells are the relatively low toxicity and lack of graft-versus-host diseases (GVHD) response. The most common side effects in CIK cell therapy are fever, chills, fatigue, headache, and skin rash, and patients can usually recover with simple treatment [26].

## 4. Clinical Studies of CIK Cells in Lymphoma Treatment

Since CIK cell therapy has been shown to be safe, there have been 17 clinical trials conducted for the treatment of lymphoma (Table 2). PubMed and Web of Science (ISI) were searched to collect all the studies based on CIK cells.

### 4.1. Clinical Studies on Autologous CIK Cells

The very first clinical trial of CIK cells on lymphoma was reported by Schmidt-Wolf et al. in 1999 [31], where 10 patients including two with FL were enrolled. The study reported that IL-2-transfected autologous CIK cells were detectable in peripheral blood up to two weeks post-infusion (Figure 1). A significant increase in IFN-γ, granulocyte-macrophage colony-stimulating factor (GM-CSF), and transforming growth factor beta (TGF-β) was also detected in the serum of patients. In terms of adverse effects, only grade 2 fever was observed in three patients during treatment, thereby demonstrating the safety and clinical potential of CIK cell therapy. Five years later, another phase I study enrolled with nine patients (seven HL, two NHL) having relapsed after autologous transplantation was reported [32]. Specifically, patients were administered with an escalating dose of CIK cells, ranging from 1 × 10^9^ to 1 × 10^10^ per dose. As a result, two patients showed partial response (PR) while two had stable disease (SD) with minimal toxicities associated with CIK cell infusion, demonstrating the feasibility of treating high-risk patients with CIK cells. In 2009, Olioso et al. conducted a pilot clinical trial with autologous CIK cells in patients with refractory lymphoma and metastatic solid tumors [33]. A total of 12 patients (four NHL and one HL) were infused with a median CIK cell count of 28 × 10^9^ per patient, and clinical outcomes were encouraging with three complete response (CR) and two SD instances. The toxicity profile was also favorable, with only grade 2 fever and/or shivering, both of which resolved promptly without the need for antibiotic treatment.

Following the success of previous studies, four independent trials were published in the year 2011 alone. Among them, Guo et al. took the initiative to infuse > 5 × 10^9^ CIK cells per treatment, which was repeated every 3–6 months in the first year and every 6 months in the second year [34]. Subsequently, the patients showed measurable radiographic reduction in tumor size by positron emission tomography-computed tomography (PET-CT), improvement in physical conditions (sleep, physical strength, appetite) and mild side effects. Considering that age is a risk factor for cancer treatments, Lu et al. specifically focused on elderly patients with DLBCL [35] and observed that two patients were in CR and seven in PR pre-treatment, while all patients achieved CR post-CIK cell infusion without any severe side effects. In the same year (2011), Yang et al. included elderly patients and confirmed the feasibility of CIK cell therapy in older patients with HL and NHL [36]. More recently, Zhou et al. investigated the efficacy and safety of autologous CIK cell treatment as maintenance therapy in patients with high-risk DLBCL after initial CR [37] and confirmed it to be safe and effective.

### 4.2. Clinical Studies on DC-CIK Cells

It is now well established that co-culture of dendritic cells (DCs) with CIK cells results in a substantial increase in anti-tumor immunity and exhibits more potent cytotoxic activity compared to CIK treatment alone [48,49]. This, in turn, also raises the possibility of DC-CIK therapy in different malignancies, including lymphomas (Figure 1). Qiu et al. (2016) showed that the combination of CIK cells and DCs pulsed with an antigenic α-1,3-galactosyl epitope-enhanced lymphoma cell membrane could provide an effective immunotherapy for B-cell lymphomas [44]. Chen et al. (2016) also evaluate the clinical efficacy of DC-CIK cell therapy in patients with advanced cancers, including lymphoma [45]. The study reported that DC-CIK infusions were able to positively alter the ratios of T-cell subsets by increasing the CD3+CD8+ (cytotoxic T cells, CTL) and CD3+CD56+ (NKT) subsets, and decreasing the CD4+CD25+ subsets without serious side effects.

### 4.3. Clinical Studies on Allogeneic CIK Cells after Allo-HSCT

Previously, it was discussed that adoptive transfer of allogeneic CIK cells in a murine model may have minor impact on graft-versus-host disease (GVHD) [50]. To pronounce the safety of these cells in allogeneic settings, Introna et al. (2007) performed a phase I study of allogeneic (donor’s) CIK cells in eleven patients relapsing after allogeneic hematopoietic stem cell transplantation (HSCT) [38]. In this study, grade I and II acute GVHD was observed in four patients, whereas extensive chronic GVHD developed in two cases. The same group then conducted a multi-center phase IIA study to test the clinical efficacy of sequential infusion of donor lymphocytes (DLI) and CIK cells in patients who had relapsed after allo-HSCT, and further confirmed the low GVHD potential and favorable safety profile of CIK cell therapy [46]. Laport et al. also observed a low incidence of GVHD, while conducting a study in patients with recurrent hematologic malignancies after allogeneic hematopoietic cell transplantation, and suggested further investigation to improve graft-versus-tumor response in high-risk patients [40]. Linn et al. (2012) and Luo et al. (2016) also reported that acute GVHD occurred in three and two patients individually, but both were controlled easily [41,43]. To mention, a previous study showed that the percentage of CD3−CD56+ cells was significantly decreased in patients with acute GVHD [51]. In CIK cells, the alloreactivity versus HLA-mismatched PBMC was restricted to the CD3+CD56− cells [52]. These results indicate that depletion of CD3+CD56− cells might further reduce the GVHD risk of CIK cells.

## 5. Improving CIK Cell Therapy on Lymphoma

**Figure 2:**
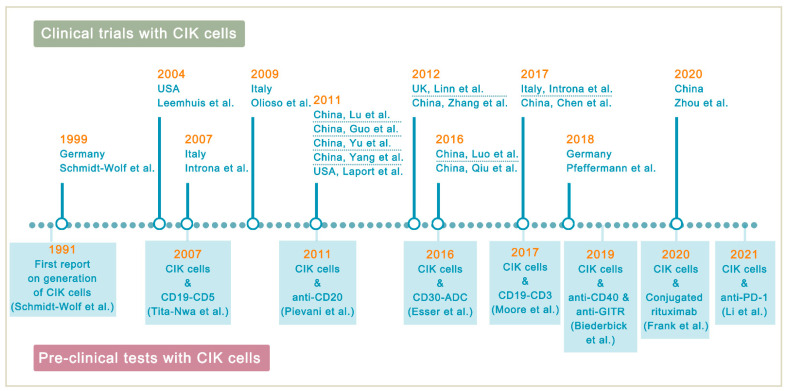
Highlights in the development of CIK cell immunotherapy. In 1991, the development of CIK therapy began (in Germany) and continued in several countries, with 2011 being the year with the most clinical trial reports. The supporting pre-clinical models that are used to improve the therapeutic activity of CIK cells are also highlighted.

Despite the positive clinical benefit observed in trials, it is difficult to achieve long-lasting response and complete cancer eradication in patients with refractory or relapsed lymphoma. Therefore, a number of pre-clinical approaches are being investigated to improve CIK cell therapy to enhance its anti-tumor activity (Figure 2). Among these innovative methods, bispecific antibodies (bsAb) represent a promising development bringing targeted antigen on tumor cells into close proximity to receptors on cytotoxic T-cells, and thereby triggering T-cell receptor signaling and anti-tumor immune response [53]. In a particular study, it was reported that the bsAb CD19 × CD5 (HD37 × T5.16) enhanced the cytotoxicity activity of CIK cells against B lymphoma cells [54]. Moore et al. used a bispecific antibody platform known as dual affinity retargeting (DART) to eradicate B-cell lymphomas by targeting the B-cell-specific antigen CD19 and the TCR/CD3 complex to effector T cells [55]. Subsequently, it was shown that CIK cells and CD19xCD3 DART can control and/or eradicate patient-derived tumor xenografts from chemo-refractory B-ALL and diffuse large B-cell lymphoma (DLBCL) patients [56].

Likewise, studies also have shown an enhancement of CIK cell activity when combined with anti-CD20 antibodies. For instance, Pievani et al. showed that the addition of the anti-CD20 antibodies GA101 or rituximab in B-NHL increased the cytotoxicity of CIK cells by 35% and 15%, respectively [57]. The authors also suggested that activation of the MAPK pathway may be a possible mechanism for the anti-apoptosis effect on CIK cell proliferation. Esser et al., investigated the effect of CIK cells in combination with brentuximab vedotin (SGN-35) on three different CD30+ lymphoma cell lines (Daudi, KI-JK, and L-540), and was found that the combined approach led to better results in vitro [58]. Recently, a novel antibody-cell conjugation method for the enhancement and characterization of cytokine-induced killer cells has been presented [59]. The authors demonstrated that CIK cells conjugated with rituximab exhibited increased cytotoxic activity against CD20+ lymphoma cell lines and suggested that without any genetic modification, CIK cells can be rapidly equipped with monoclonal antibodies to target tumor cells.

Biederbick et al. recently contributed to raise concerns about synergistic molecular mechanisms of CIK cells by using a combination of anti-CD40 and anti-GITR mAb in the human lymphoma cell lines SU-DHL-4 and Daudi (both CD40-positive) [60]. More recently, Li et al. reported an increase in IFN-γ secretion in B-NHL cell lines treated with CIK alone or with the PD-1 antibody, yet this trend was not observed for PD-L1, raising the question of whether PD-1 and PD-L1 are comparable and interchangeable in the clinical practice [61].

## 6. Conclusions

CIK cells have demonstrated favorable safety profile and promising anti-tumor response in the 17 lymphoma-specific clinical trials. The improvements on its cytotoxicity will further make CIK cells an important tool in future therapeutic regimens. As GVHD is one of the most severe complications in donor lymphocyte infusion (DLI) after HSCT, CIK cells would be a favorable alternative to DLI due to its low allo-reactive potential. Besides, patients with central nervous system (CNS) pathology and declining performance status are usually not suitable for CAR-T therapy. CIK cells with mild toxicities would be one of the feasible options for the lymphoma patients after receiving two prior lines of therapy.

Considering that each lymphoma patient could benefit from individual therapy (as a personalized therapeutic approach), it is therefore important to stratify those subgroups that respond well to CIK cell therapy, and to understand the precise molecular mechanisms that support the functioning CIK cells in their disease state. As an initiative, the International Registry for CIK Cells (IRCC) has been established to collect clinical data worldwide and to set new standards for global reporting of clinical trials involving CIK cells [26,62,63]. On molecular grounds, lymphomas being heterogenous in nature may help to elucidate different factors (genetic, epigenetic, microenvironment, immunological etc.) that can improve the therapeutic effects of CIK cells. On a broader prospective, it can be concluded that CIK cell therapy has potential and in this rapidly evolving landscape of cancer therapies, optimizing CIK cell therapy in combination with other modules (as complementary rather than competitive) will be a crucial task in lymphomas.

## Figures and Tables

**Table 1:** Similarities and differences between CIK cells, CAR-T cells and BiTEs in lymphoma.

	CIK Cells	CAR-T Cells	BiTEs
Manufacturing Process	PBMCs sequentially activated with 1000 IU/m IFN-γ on day 0, and 50 ng/mL anti-CD3 mAb and 600 IU/mL IL-2 on the following day; IL-2 supplemented every 2–3 days	T cells with CAR gene transduction primarily by lentiviruses; CARs consist of an scFv based ectodomain for antigen-binding, a transmembrane domain, and an endodomain containing TCR CD3ζ chain with or without costimulatory signaling components	Antibodies designed to bind to a selected TAA and CD3 on T cells simultaneously; produced in bioreactors by mammalian cell lines as secreted polypeptides
Effector Cells	CD3+CD56+ cells	Mostly αβ-TCR+ T cells	Endogenous CD8+ or CD4+ T cells
Cell Source	Autologous/allogeneic	Autologous/allogeneic	Autologous
Target Antigen	MIC A/B and ULBP1–4	CD19, CD20, CD22, CD30, BCMA, etc.	CD19
MHC Restriction	Dual-functional capability (non-MHC-restricted and TCR-mediated lysis)	TAA recognition by CARs is MHC-unrestricted	MHC-unrestricted
Mechanism	Release of perforin and granzyme B from CIK cells	Release of perforin and granzyme B from CAR-T cells	Activating T cells to release perforin and granzyme B by linking TAAs to CD3 on T cells
Toxicities and Side effects	Low-grade toxicities including fever, chills, fatigue, headache, and skin rash; grade 3 and 4 toxicities are rare; limited GVHD response in the allogeneic setting	CRS, ICANS, and MAS/HLH; potentially life-threatening	CRS and ICANS; severe toxicities are one of the major concerns
Clinical Efficacy	Varied due to heterogeneity of expension method and study design	Axicabtagene ciloleucel: 83% ORR, 58% CR; Tisagenlecleucel: 54% ORR, 40% CR; Lisocabtagene maraleucel: 73% ORR, 53% CR [27,28,29]	Blinatumomab: 36% to 69% ORR in relapsed/refractory NHL [11,30]

CIK cells, cytokine-induced killer cells; MIC A/B, MHC class I-related molecules A and B; ULBP1–4, UL16-binding protein; GVHD, graft-versus-host diseases; CAR, chimeric antigen receptors; scFv, single-chain variable fragment; TCR, T cell receptor; TAA, tumor-associated antigen; CRS, cytokine release syndrome; ICANS, immune effector cell-associated neurotoxicity syndrome; MAS/HLH, Macrophage activation syndrome/hemophagocytic lymphohistiocytosis; CR, complete remission; ORR, objective response rate; BiTE, bispecific T cell engager.

**Table 2:** Clinical trials based on CIK cell therapy in lymphoma.

Study	Phase	Number of Patients	Therapeutic Approach	Clinical Response	Adverse Event	Conclusion
Schmidt-Wolf et al. (1999) [31]	Phase I	10 (2 FL)	IL-2-transfected + untransfected auto-CIKs	1 CR (1 FL), 6 PD; increased alkaline phosphatase and CRP	Grade 2 fever but resolved the next day with or without addition of antibiotics	CIK cells transfected with IL-2 gene can be administered without major side effects
Leemhuis et al. (2004) [32]	Phase I	9 (7 HL, 2 NHL)	Auto-CIKs	2 PR, 2 SD	1 case of asymptomatic mild hypotension that quickly resolved; fever (38 °C); 1 patient with thrombocytopenia (thought to be related to disease progression)	CIK cells may have utility for the treatment of high-risk patients with evidence of minimal residual disease after autologous transplantation
Introna et al. (2007) [38]	Phase I	11 (3 HL)	Allo-CIKs	3 CR (1 HL), 1 SD (1 HL), 6 PD or death	Acute grade I and II GVHD in 4 patients	Allogeneic CIK cells are well tolerated and may contribute to clinical responses in patients relapsing after allogeneic HSCT
Olioso et al. (2009) [33]	Pilot	12 (1 FL, 1 B-cell NHL, 1 DLBCL, 1 HL, 1 centroblastic-centrocytic NHL)	Auto-CIKs	3 CR (1 NHL), 2 SD	Graded 2 fever and/or chills during the first cycle of infusions (4.3%) but promptly resolved without antibiotic treatment	CIK cell therapy is a safe treatment with some suggestion of efficacy that significantly enhances immune functions increasing absolute numbers of effector cells
Lu et al. (2011) [35]	NA	9 (9 DLBCL)	Auto-CIKs + rhIL-2 subcutaneously administered	9 CR; serum levels of β2-microglobulin and LDH were significantly decreased; higher Karnofsky score	2 patients developed mild fatigue and low-grade fever at the initialadministration of rhIL-2	Autologous CIK cell therapy is safe and efficacious for the treatment of elderly patients with DLBCL
Guo et al. (2011) [34]	NA	8 (2 HL, 6 NHL)	Auto-CIKs	1 CR, 7 PR	Fever (38 °C) resolved with or without treatment, headache, nausea, vomiting, irritation disappearing after treatment withdrawal	CIK cell treatment of patients with refractory lymphoma achieves effective clinical responses with few side effects
Yu et al. (2011) [39]	NA	20 (1 NHL)	Auto-CIKs + CH-296	12 SD, 8 PD (1 NHL) or death; 1-year survival rate 65%; mean OS 16.95 ± 6.10 months	Transient fever	CH-296 and IFN-γ synergistically promote anti-tumor efficiency of CIKs in advanced-stage malignant tumors
Yang et al. (2011) [36]	Pilot	20 (1 LPL, 1 HL, 2 gastric MALT lymphoma, 5 DLBCL, 3CLL)	Auto-CIKs + subcutaneous injection of rhIL-2	11 CR (1 LPL, 1 HL, 2 Gastric MALT lymphoma, 2 DLBCL, 2 CLL), 7 PR (3 DLBCL, 1 CLL), 2 SD; mean survival time 20 months; increased Karnofsky score	Mild malaise and low-grade fever	Autologous CIK cells plus rhIL-2 treatment is safe and effective for treating haematological malignancies in elderly patients
Laport et al. (2011) [40]	NA	18 (7 NHL, 1 HL)	Allo-CIKs	Mean OS 28 months; mean event-free survival 4 months; 12 CR (5 NHL, 1 HL), 3 PR (2 CLL), 3 PD	2 patients with acute GVHD resolved with corticosteroids; 1 patient with chronic GVHD; transient grade 3 ventricular arrhythmia; transient rise in hepatic transaminase levels	CIK cell treatment is well tolerated and induces a low incidence of GVHD in patients with relapsed hematologic malignancies after allogeneic HSCT
Linn et al. (2012) [41]	Phase I/II	16 (3 HL, 1 NHL)	Allo-CIKs	5 patients achieved response attributable to CIK cell infusion (1 HL)	Acute GVHD occurred in three but easily treatable	This study provides some evidence suggestive of the efficacy of allogeneic CIK cells even after failure of DLI
Zhang et al. (2012) [42]	Retrospective	40 (1 DLBCL)	Auto-CIKs	The 6-month, 1-year and 3-year OS rates were 70.0, 60.0 and 57.5%, respectively; The median OS was 34.9 months	Fever and poor appetite relieved quickly by simple treatment	CIKs immunotherapy can be an effective adjuvant instrument of the routine therapy of malignancy
Luo et al. (2016) [43]	Phase I	15 (1 SLL, 1 ALCL, 2 DLBCL)	Allo-CIKs	All patients achieved engraftment and CR; 9 patients had died and 6 patients were surviving at a median follow-up of 1513 days	Two patients developed GVHD (grade I and III) but were controlled by additional immunosuppressant drugs; grade IV neutropenia in the transplant phase	Allogeneic HSCT combined with sequential infusion of donor CIK cells is well tolerated in salvage relapsed/refractory hematologic malignancy patients
Qiu et al. (2016) [44]	NA	14 (4 FL, 7 DLBCL, 1 NMZL, 1 ALCL, 1 LBL)	Auto-CIKs + dendritic cells pulsed with antigenic α-1,3-galactosyl epitope	4 CR, 3 PR, 2 PD; Karnofsky score improved dramatically in 12 of 14 patients	Fever (37.5–38.0 °C), skin rash, transient hypotension	Combination of CIK and dendritic cells pulsed with antigenic α-1,3-galactosyl epitope is safe and has great potential for the treatment of refractory B-cell lymphoma
Chen et al. (2017) [45]	NA	90 (8 lymphoma)	Group 1: Auto-CIKs + dendritic cells Group 2: Best supportive care alone	OS of patients in CIK and dendritic cell group was significantly higher than best supportive care group (14 months vs. 11 months)	Fever (<38.5 °C) spontaneously relieved	CIK and dendritic cell therapy can improve the imbalance of immune system and prolong the OS in patients with advanced cancer
Introna et al. (2017) [46]	Phase IIA	74 (3 HL, 2 NHL)	DLI + Allo-CIKs	19 CR, 3 PR, 8 SD, 41 PD, 2 death; 1-year and 3-year PFS was 31% and 29%; 1-year and 3-year was 51% and 40%	12 patients (16%) developed acute GVHD (grade I–II in 7 cases and grade III–IV in 5); 11 patients (15%) developed chronic GVHD	A low incidence of GVHD is observed after the sequential administration of DLI and CIK cells and disease control can be achieved mostly after a cytogenetic or molecular relapse
Pfeffermann et al. (2018) [47]	NA	1 PTLD	Allo-CIKs with EBV-specificity	Long-term clearance of plasma EBV DNA and rapid and sustained disappearance of large DLBCL nodes within 7 days	Chronic GVHD of the skin 6 months after CIK cell treatment improved with immunosuppressive medication	EBV peptide-induced CIK cells might be considered a therapy for EBV-related PTLD
Zhou et al. (2020) [37]	NA	40 (40 DLBCL)	Group 1: Auto-CIKs Group 2: No transfusion	Significantly improved 5-year DFS of CIK group (79.3 ± 9.2% vs. 45.0 ± 11.1%) and 5-year OS of CIK group (90 ± 6.7% vs. 55 ± 11.1%)	Mild flu-like symptoms that was quickly relieved	Autologous CIK cell immunotherapy is a safe and efficacious option to improve the prognosis of patients with high-risk DLBCL after the first CR

CR, complete response; PR, partial response; PD, progression disease; SD, stable disease; PFS, progression-free survival; OS, overall survival; Auto, autologous; Allo, allogeneic.
